# Supplementary material for: Compliance with COVID-19 government guidance and rules by disabled people and people from minoritised ethnic groups: Qualitative findings from the CICADA study
Source: PLoS One. 2024 Sep 19;19(9):e0301242. doi: 10.1371/journal.pone.0301242 (PMC11412500; doi:10.1371/journal.pone.0301242)
Supplement: S1 Table — Tables a-e provide a ‘big picture’ quantitative content analysis of compliance by ethnic group, disability category and site, and comparison with non-core participants (the sensitivity analysis). The ‘unit of analysis’ is individual people. In other words, the data represent the proportion of people in each ethnic, disability, or regional group (the rows) for whom each feature (i.e. column label) was true. Percentages are rounded to two significant figures. Low percentages for a theme may simply indicate lack of response, therefore these tables are only indicative of the distribution of responses across groups. However sample sizes were sufficiently large to have some confidence in findings. Tables are Excel-generated ‘heat maps’, with blue shades representing low percentages and red high ones. Where percentages are low, we emphasise this in the associated paper or more commonly do not comment on that particular feature for that particular group. (DOCX) [file pone.0301242.s001.docx]

**S1: Heat maps to show the relative mention of different features of compliance by ethnicity, disability and region**

Tables a-e provide a ‘big picture’ quantitative content analysis of compliance by ethnic group, disability category and site, and comparison with non-core participants (the sensitivity analysis). The ‘unit of analysis’ is individual people. In other words, the data represent the proportion of people in each ethnic, disability, or regional group (the rows) for whom each feature (i.e. column label) was true. Percentages are rounded to two significant figures. Low percentages for a theme may simply indicate lack of response, therefore these tables are only indicative of the distribution of responses across groups. However sample sizes were sufficiently large to have some confidence in findings. Tables are Excel-generated ‘heat maps’, with blue shades representing low percentages and red high ones. Where percentages are low, we emphasise this in the associated paper or more commonly do not comment on that particular feature for that particular group.

1. **Ethnicity and vaccination**

|  | |  |  | |  | | |  | | | | |  | | |  |  | |  | |  | |  |  | | | |  | | | | | | |  |  | | | | |  |  |  |  |  |  |
| --- | --- | --- | --- | --- | --- | --- | --- | --- | --- | --- | --- | --- | --- | --- | --- | --- | --- | --- | --- | --- | --- | --- | --- | --- | --- | --- | --- | --- | --- | --- | --- | --- | --- | --- | --- | --- | --- | --- | --- | --- | --- | --- | --- | --- | --- | --- |
|  | |  | % of total n | | | | | | | | | | | | | | | | | | | | | | | | | | | | | | | |  |  |  |  |  |  |  |  |  |  |  |  |
| Ethnicity group | | Total n | (Core n) | | Not taken vaccine | | | Con-sidering | | | Side effects/ other fear | | | Con-fused/. need info | | | | Need to travel/work | | Free choice issue /anti-vax | Inter-action with con-dition/treat-ment | Had covid and was ok/not dis-abled | | Trad- itional rem-edies pref-erred | Ex-perienced close COVID deaths (context, not linked to vaccine uptake) | | | | Attended COVID-19 funerals (context. not linked to vaccine uptake) | | | |  | | | |  |  |  |  |  |  |  |  |  |  |
| CORE ETHNIC GROUPS | |  | | | | | | | | | | | | | | | | | | | | | | | | | | | | | | |  | | | |  |  |  |  |  |  |  |  |  |  |
| South Asia | | 96 | 92 | | 8.33 | | | 1.04 | | | 13.54 | | | 5.21 | | | | 4.17 | | 0.00 | 1.04 | 0.00 | | 4.17 | 6.25 | | | | 4.17 | | | |  | | | |  |  |  |  |  |  |  |  |  |  |
| North Africa | | 48 | 38 | | 8.33 | | | 8.33 | | | 25.00 | | | 0.00 | | | | 0.00 | | 0.00 | 2.08 | 0.00 | | 8.33 | 4.17 | | | | 0.00 | | | |  | | | |  |  |  |  |  |  |  |  |  |  |
| Other Arab League | | 24 | 19 | | 4.17 | | | 0.00 | | | 29.17 | | | 0.00 | | | | 0.00 | | 0.00 | 0.00 | 0.00 | | 4.17 | 0.00 | | | | 0.00 | | | |  | | | |  |  |  |  |  |  |  |  |  |  |
| Central and East Europe | | 27 | 22 | | 18.52 | | | 11.11 | | | 11.11 | | | 11.11 | | | | 22.22 | | 11.11 | 3.70 | 3.70 | | 7.41 | 0.00 | | | | 0.00 | | | |  | | | |  |  |  |  |  |  |  |  |  |  |
| African | | 30 | 25 | | 3.33 | | | 13.33 | | | 20.00 | | | 0.00 | | | | 6.67 | | 0.00 | 3.33 | 0.00 | | 15.15 | 3.03 | | | | 3.03 | | | |  | | | |  |  |  |  |  |  |  |  |  |  |
| undocumented (more were included but did not want to be so identified) | | 6 | 6 | | 16.67 | | | 0.00 | | | 16.67 | | | 0.00 | | | | 0.00 | | 0.00 | 0.00 | 0.00 | | 50.00 | 0.00 | | | | 0.00 | | | |  | | | |  |  |  |  |  |  |  |  |  |  |
| **CORE ethnic groups total** | | **231** | **202** | | **8.66** | | | **5.19** | | | **18.18** | | | **3.46** | | | | **5.19** | | **1.30** | **1.73** | **0.43** | | **8.23** | **3.90** | | | | **2.16** | | | |  | | | |  |  |  |  |  |  |  |  |  |  |
| NATIVE WHITE BRITISH | | **20** | **16** | | **0.00** | | | **0.00** | | | **5.00** | | | **0.00** | | | | **5.00** | | **0.00** | **0.00** | **0.00** | | **0.00** | **0.00** | | | | **0.00** | | | |  |  |  |  |  |  |  |  |  |  |  |  |  |  |
| OTHER ETHNIC GROUPS | |  | | | | | | | | | | | | | | | | | | | | | | | | | | | | | | | |  | | | | | | | | |  | | | |
| Caribbean | | 2 | 0 | | | 50.00 | | | 0.00 | | | 0.00 | | | 0.00 | | | 0.00 | | 0.00 | 0.00 | 0.00 | | 0.00 | 0.00 | | | | | 0.00 | | | |  | | | | | | | | |  | | | |
| South Europe | | 9 | 0 | | | 0.00 | | | 0.00 | | | 11.11 | | | 0.00 | | | 22.22 | | 0.00 | 0.00 | 0.00 | | 0.00 | 0.00 | | | | | 0.00 | | | |  | | | | | | | | |  | | | |
| Mixed Race | | 9 | 0 | | | 11.11 | | | 0.00 | | | 0.00 | | | 22.22. | | | 0.00 | | 11.11 | 0.00 | 0.00 | | 11.11 | 0.00 | | | | | 0.00 | | | |  | | | | | | | | |  | | | |
| **Other ethnic groups total** | | **20** | **0** | | | **10.00** | | | **0.00** | | | **5.00** | | | **10.00** | | | **10.00** | | **5.00** | **5.00** | **0.00** | | **5.00** | **0.00** | | | | | **0.00** | | | |  | | | | | | | | |  | | | |
| **b) Disability and vaccination (sensitivity analysis: data poor for subthemes for non-disabled).** | | | | | | | | | | | | | | | | | | | | | |  | |  |  | | | | |  | | | | | | | | |  | | | | |  |  |  |
|  | |  | % of total n | | | | | | | | | | | | | | | | | | | | | | | | | | | |  | | | | | | |  | | | |  |  |  |  |  |
| Disability/ condition groups | | Total n | Core n | | | Not taken vaccine | | | Con-sidering | | | Side effects/ other fear | | | Con-fused/. need info | | | Need to travel/work | | Free choice issue /anti-vax | Inte-raction with con-dition/ treat-ment | Had covid and was ok/not dis-abled | | Experienced close COVID deaths (context, not linked to vaccine uptake) | | Attended COVID-19 funerals (context. not linked to vaccine uptake) | | | | |  | | | | | | |  | | | |  |  |  |  |  |
| Dexterity | | 3 | 3 | | | 0.00 | | | 33.33 | | | 0.00 | | | 0.00 | | | 0.00 | | 0.00 | 0.00 | 0.00 | | 0.00 | | 0.00 | | | | |  | | | | | | |  | | | |  |  |  |  |  |
| Mental Health | | 19 | 18 | | | 21.05 | | | 0.00 | | | 21.05 | | | 10.53 | | | 26.32 | | 5.26 | 0.00 | 0.00 | | 0.00 | | 0.00 | | | | |  | | | | | | |  | | | |  |  |  |  |  |
| Mobility | | 35 | 34 | | | 0.00 | | | 8.57 | | | 20.00 | | | 0.00 | | | 11.43 | | 0.00 | 2.86 | 0.00 | | 5.71 | | 2.86 | | | | |  | | | | | | |  | | | |  |  |  |  |  |
| Neuro-divergence | | 5 | 5 | | | 20.00 | | | 0.00 | | | 20.00 | | | 0.00 | | | 20.00 | | 0.00 | 0.00 | 0.00 | | 0.00 | | 0.00 | | | | |  | | | | | | |  | | | |  |  |  |  |  |
| Cognitive | | 4 | 4 | | | 0.00 | | | 0.00 | | | 0.00 | | | 0.00 | | | 0.00 | | 0.00 | 0.00 | 0.00 | | 0.00 | | 0.00 | | | | |  | | | | | | |  | | | |  |  |  |  |  |
| Brain hyper-excitability (migraines, epilepsy) | | 7 | 6 | | | 14.29 | | | 0.00 | | | 14.29 | | | 14.29 | | | 14.29 | | 0.00 | 0.00 | 0.00 | | 0.00 | | 0.00 | | | | |  | | | | | | |  | | | |  |  |  |  |  |
| Food-relevant | | 18 | 17 | | | 11.11 | | | 5.56 | | | 22.22 | | | 5.56 | | | 5.56 | | 0.00 | 5.56 | 0.00 | | 0.00 | | 0.00 | | | | |  | | | | | | |  | | | |  |  |  |  |  |
| Sensorial | | 5 | 3 | | | 20.00 | | | 0.00 | | | 20.00 | | | 20.00 | | | 20.00 | | 0.00 | 0.00 | 0.00 | | 0.00 | | 0.00 | | | | |  | | | | | | |  | | | |  |  |  |  |  |
| Cancer | | 14 | 10 | | | 7.14 | | | 7.14 | | | 21.43 | | | 0.00 | | | 0.00 | | 0.00 | 7.14 | 0.00 | | 0.00 | | 0.00 | | | | |  | | | | | | |  | | | |  |  |  |  |  |
| Stamina | | 44 | 34 | | | 6.82 | | | 6.82 | | | 15.91 | | | 2.27 | | | 0.00 | | 2.27 | 2.27 | 2.27 | | 2.27 | | 0.00 | | | | |  | | | | | | |  | | | |  |  |  |  |  |
| 2 conditions | | 66 | 63 | | | 9.09 | | | 4.55 | | | 10.61 | | | 3.03 | | | 6.06 | | 3.03 | 1.52 | 0.00 | | 4.55 | | 7.58 | | | | |  | | | | | | |  | | | |  |  |  |  |  |
| 3+ conditions | | 25 | 21 | | | 8.00 | | | 0.00 | | | 8.00 | | | 8.00 | | | 4.00 | | 0.00 | 0.00 | 0.00 | | 12.00 | | 4.00 | | | | |  | | | | | | |  | | | |  |  |  |  |  |
| **All disabilities total** | | **245** | **218** | | | 8.57 | | | 4.90 | | | 15.10 | | | 4.08 | | | 7.35 | | 1.63 | 2.04 | 0.41 | | 3.67 | | 2.86 | | | | |  | | | | | | |  | | | |  |  |  |  |  |
| No disabilities/ conditions | | 20 | - | | | 5.00 | | | 0.00 | | | 30.00 | | | 0.00 | | | 10.00 | | 0.00 | 0.00 | 0.00 | | 0.00 | | 0.00 | | | | |  | | | | | | |  | | | |  |  |  |  |  |
| Conditions not disabling (sensitivity analysis) | | 6 |  | | | 0.00 | | | 0.00 | | | 16.67 | | | 0.00 | | | 0.00 | | 0.00 | 0.00 | 0.00 | | 0.00 | | 0.00 | | | | |  | | | | | | |  | | | |  |  |  |  |  |
| **No disabilities total** | | **26** | **0** | | | **3.85** | | | **0.00** | | | **26.92** | | | **0.00** | | | **7.69** | | **0.00** | **0.00** | **0.00** | | **0.00** | | **0.00** | | | | |  | | | | | | |  | | | |  |  |  |  |  |
| **Multimorbidities** | | 91 | 84 | | | 8.79 | | | 3.30 | | | 9.89 | | | 4.40 | | | 5.49 | | 2.20 | 1.10 | 0.00 | | 6.59 | | 6.59 | | | | |  | | | | | | |  | | | |  |  |  |  |  |
| **c) Ethnicity and compliance** | | | | | | | | | | | | | | | | | | | | | | | |  | |  | | | | |  | | | | | | |  | | | |  |  |  |  |  |
|  | |  |  | | |  | | |  | | |  | | |  | | |  | |  |  |  | |  | |  | | | | |  | | | | | | |  | | | |  |  |  |  |  |
|  | |  | % of total n | | | | | | | | | | | | | | | | | | | | |  | |  | | | | |  | | | | | | |  | | | |  |  |  |  |  |
| Ethnicity group | | Total n | Com-plied | | | Part com-plied | | | Did not comply | | | Feared others | | | Pro government | | | Anti-government | | Un-sure of gov-ern-ment | Mask issues | Mask not worn | |  | |  | | | | |  | | | | | | |  | | | |  |  |  |  |  |
| CORE ETHNIC GROUPS | |  | | | | | | | | | | | | | | | | | | | | | |  | |  | | | | |  | | | | | | |  | | | |  |  |  |  |  |
| South Asia | | 96 | 69.79 | | | 14.58 | | | 2.08 | | | 20.83 | | | 13.54 | | | 34.38 | | 0.00 | 21.88 | 4.17 | |  | |  | | | | |  | | | | | | |  | | | |  |  |  |  |  |
| North Africa | | 48 | 79.17 | | | 6.25 | | | 0.00 | | | 6.25 | | | 12.50 | | | 16.67 | | 6.25 | 20.83 | 0.00 | |  | |  | | | | |  | | | | | | |  | | | |  |  |  |  |  |
| Other Arab League | | 24 | 95.83 | | | 0.00 | | | 4.17 | | | 16.67 | | | 12.50 | | | 20.83 | | 4.17 | 20.83 | 0.00 | |  | |  | | | | |  | | | | | | |  | | | |  |  |  |  |  |
| Central and East Europe | | 27 | 37.04 | | | 25.93 | | | 0.00 | | | 7.41 | | | 7.41 | | | 40.74 | | 7.41 | 11.11 | 0.00 | |  | |  | | | | |  | | | | | | |  | | | |  |  |  |  |  |
| Africa | | 30 | 13.33 | | | 0.00 | | | 6.67 | | | 3.33 | | | 26.67 | | | 26.67 | | 0.00 | 13.33 | 0.00 | |  | |  | | | | |  | | | | | | |  | | | |  |  |  |  |  |
| undocumented (more were included but did not want to be so identified) | | 6 | 100.00 | | | 0.00 | | | 0.00 | | | 33.33 | | | 16.67 | | | 33.33 | | 0.00 | 33.33 | 0.00 | |  | |  | | | | |  | | | | | | |  | | | |  |  |  |  |  |
| **CORE ethnic groups total** | | **231** | 75.76 | | | 10.39 | | | 2.16 | | | 13.85 | | | 14.29 | | | 29.00 | | 2.60 | 19.48 | 1.73 | |  | |  | | | | |  | | | | | | |  | | | |  |  |  |  |  |
| NATIVE WHITE BRITISH | | 20 | 55.00 | | | 10.00 | | | 10.00 | | | 20.00 | | | 0.00 | | | 35.00 | | 0.00 | 10.00 | 0.00 | |  | |  | | | | |  | | | | | | |  | | | |  |  |  |  |  |
| OTHER ETHNIC GROUPS | | | | | | | | | | | | | | | | | | | | | | | |  | | |  | | | | | | | |  | | | | |  | | | | | |  |
| Caribbean | 2 | | | 100.00 | | | 0.00 | | | 0.00 | | 0.00 | | | 0.00 | | | 100.00 | | 0.00 | 0.00 | 0.00 | |  | | | | | | | |  | | | | |  | | | | | | | |  | |
| South Europe | 9 | | | 66.67 | | | 0.00 | | | 0.00 | | 11.11 | | | 22.22 | | | 33.33 | | 0.00 | 22.22 | 0.00 | |  | | | | | | | |  | | | | |  | | | | | | | |  | |
| Mixed Race | 9 | | | 100.00 | | | 0.00 | | | 0.00 | | 11.11 | | | 0.00 | | | 55.56 | | 0.00 | 22.22 | 0.00 | |  | | | | | | | |  | | | | |  | | | | | | | |  | |
| **Other ethnic groups total** | **20** | | | **85.00** | | | **0.00** | | | **0.00** | | **10.00** | | | **10.00** | | | **50.00** | | **0.00** | **20.00** | **0.00** | |  | | | | | | | |  | | | | |  | | | | | | | |  | |

d) Disability and compliance

|  |  |  |  | |  | |  | |  | |  | |  | |  | |  | |  |  |  |  |  |  |  |  |
| --- | --- | --- | --- | --- | --- | --- | --- | --- | --- | --- | --- | --- | --- | --- | --- | --- | --- | --- | --- | --- | --- | --- | --- | --- | --- | --- |
| Disability/ condition groups | N | Com-plied | | Did not com-ply | | Feared others | | Pro government | | Anti-government | | Unsure about govern ment | | Felt lonely | | Mask issues | | Forgot mask | |  |  |  |  |  |  |  |
| Dexterity | 3 | 100.00 | | 0.00 | | 0.00 | | 0.00 | | 0.00 | | 0.00 | | 0.00 | | 100.00 | | 0.00 | |  |  |  |  |  |  |  |
| Mental Health | 19 | 94.74 | | 0.00 | | 21.05 | | 0.00 | | 36.84 | | 0.00 | | 31.58 | | 15.79 | | 5.26 | |  |  |  |  |  |  |  |
| Mobility | 35 | 88.57 | | 2.86 | | 11.43 | | 11.43 | | 37.14 | | 0.00 | | 14.29 | | 14.29 | | 0.00 | |  |  |  |  |  |  |  |
| Neuro-divergence | 5 | 60.00 | | 0.00 | | 0.00 | | 20.00 | | 40.00 | | 0.00 | | 20.00 | | 20.00 | | 0.00 | |  |  |  |  |  |  |  |
| Cognitive | 4 | 0.00 | | 0.00 | | 0.00 | | 0.00 | | 0.00 | | 0.00 | | 0.00 | | 0.00 | | 0.00 | |  |  |  |  |  |  |  |
| Brain hyper-excitability (migraines, epilepsy) | 7 | 85.71 | | 0.00 | | 42.86 | | 28.57 | | 42.86 | | 0.00 | | 14.29 | | 42.86 | | 0.00 | |  |  |  |  |  |  |  |
| Food-relevant | 18 | 94.44 | | 0.00 | | 5.56 | | 16.67 | | 33.33 | | 0.00 | | 16.67 | | 5.56 | | 0.00 | |  |  |  |  |  |  |  |
| Sensorial | 5 | 40.00 | | 40.00 | | 0.00 | | 20.00 | | 40.00 | | 0.00 | | 40.00 | | 0.00 | | 0.00 | |  |  |  |  |  |  |  |
| Cancer | 14 | 78.57 | | 0.00 | | 7.14 | | 28.57 | | 42.86 | | 0.00 | | 21.43 | | 28.57 | | 0.00 | |  |  |  |  |  |  |  |
| Stamina | 44 | 75.00 | | 0.00 | | 11.36 | | 15.91 | | 27.27 | | 2.27 | | 13.64 | | 18.18 | | 0.00 | |  |  |  |  |  |  |  |
| 2 conditions | 66 | 17.58 | | 3.03 | | 18.18 | | 10.61 | | 30.30 | | 4.55 | | 21.21 | | 24.24 | | 0.00 | |  |  |  |  |  |  |  |
| 3+ conditions | 25 | 88.00 | | 8.00 | | 12.00 | | 8.00 | | 48.00 | | 0.00 | | 28.00 | | 16.00 | | 0.00 | |  |  |  |  |  |  |  |
| **All disabilities total** | **245** | 88.98 | | 2.86 | | 13.47 | | 12.65 | | 33.88 | | 1.63 | | 19.59 | | 19.59 | | 0.41 | |  |  |  |  |  |  |  |
| **Not disabled Total** | 20 | 100.00 | | 0.00 | | 20.00 | | 15.00 | | 15.00 | | 0.00 | | 5.00 | | 10.00 | | 0.00 | |  |  |  |  |  |  |  |
| Multimorbid | 91 | 12.20 | | 4.40 | | 16.48 | | 9.89 | | 35.16 | | 3.30 | | 23.08 | | 21.98 | | 0.00 | |  |  |  |  |  |  |  |

| e) Sites and compliance | | | | | | | | | | | | | | | | | |
| --- | --- | --- | --- | --- | --- | --- | --- | --- | --- | --- | --- | --- | --- | --- | --- | --- | --- |
|  | N= | complied | Not complied | Pro government | Anti government | Unsure about government | anti:pro ratio | Mask issues | Mask not worn | Vaccine not taken | Considering taking vaccine | Worried about behav-iour of others | Traditional remedies preferred | Experienced close COVID deaths (context, not linked to vaccine uptake) | Attended COVID-19 funerals (context. not linked to vaccine uptake) | Felt lonely | Suicidal |
| London | 108 | 77.78 | 2.78 | 13.89 | 37.04 | 0.93 | 2.67 | 22.22 | 2.78 | 10.19 | 6.48 | 12.96 | 6.48 | 0.93 | 1.85 | 18.52 | 5.56 |
| Manchester | 36 | 75.00 | 0.00 | 5.56 | 30.56 | 2.78 | 5.50 | 16.67 | 2.78 | 2.78 | 2.78 | 16.67 | 8.33 | 0.00 | 0.00 | 22.22 | 0.00 |
| Yorks | 28 | 75.00 | 7.14 | 3.57 | 39.29 | 3.57 | 11.00 | 10.71 | 10.71 | 10.71 | 10.71 | 21.43 | 3.57 | 10.71 | 10.71 | 21.43 | 0.00 |
| Midlands | 30 | 93.33 | 0.00 | 10.00 | 30.00 | 0.00 | 3.00 | 23.33 | 0.00 | 3.33 | 3.33 | 13.33 | 10.00 | 3.33 | 0.00 | 3.33 | 0.00 |
| SE | 21 | 80.95 | 0.00 | 19.05 | 19.05 | 0.00 | 1.00 | 9.52 | 0.00 | 23.81 | 0.00 | 14.29 | 9.52 | 14.29 | 0.00 | 9.52 | 14.29 |
| Newcastle | 17 | 76.47 | 0.00 | 11.76 | 23.53 | 0.00 | 2.00 | 29.41 | 5.88 | 0.00 | 0.00 | 35.29 | 11.76 | 5.88 | 0.00 | 29.41 | 0.00 |
| **tot core** | **240** | 79.17 | 2.08 | 11.25 | 32.92 | 1.25 | 2.93 | 19.58 | 3.33 | 8.75 | 5.00 | 16.25 | 7.50 | 3.75 | 2.08 | 17.50 | 0.00 |
| Wales and Scotland | 24 | 100.00 | 0.00 | 25.00 | 16.67 | 12.50 | 0.67 | 12.50 | 0.00 | 4.17 | 0.00 | 0.00 | 12.50 | 0.00 | 0.00 | 12.50 | 0.00 |
| UnK | 1 | 0.00 | 0.00 | 100.00 | 0.00 | 0.00 | 0.00 | 0.00 | 0.00 | 0.00 | 0.00 | 0.00 | 0.00 | 0.00 | 0.00 | 0.00 | 0.00 |
