# Supplementary material for: Compliance with COVID-19 government guidance and rules by disabled people and people from minoritised ethnic groups: Qualitative findings from the CICADA study
Source: PLoS One. 2024 Sep 19;19(9):e0301242. doi: 10.1371/journal.pone.0301242 (PMC11412500; doi:10.1371/journal.pone.0301242)
Supplement: Attachment — Submitted filename: Response to Reviewers.docx [file pone.0301242.s002.docx]

Dear Reviewers

Thank you for your comments which I believe have improved the quality of the paper. Below please find my responses.

| **Reviewer 1** |  |
| --- | --- |
| This was a well written paper with a clear research purpose and interesting set of results. The findings present as a very useful historical record of the experiences of these communities during the pandemic. I thought the timeline presentation a neat way to convey the results. The discussion clearly articulated where the findings were similar to other research and the particular contributions of this research paper. | Many thanks for this |
| My main suggestion is that in places the paper could potentially be shortened to make it easier for the reader. | This has been done as per your more specific suggestions. |
| Tables 2 – 6 contained a lot of information that probably needed more narrative explanation so that reader could gleam the important points. It was hard to know how much these tables were contributing to the overall paper and whether some of this material was necessary.  There were places during the results were there was commentary comparing quant findings between different communities. In the method section it was mentioned that a survey was conducted as part of this study but only the qualitative findings were mentioned in this paper. If there are quant findings from a survey I wasn’t sure why there was a need to turn these qualitative data into quantitative data and commentary. I assume the sample sizes were too low to conduct any inferential tests so I wasn’t sure of the value of this type of analysis. At times the numbers were helpful to orient the reader to the sample characteristics. At other times there was actual comparison of loneliness, vaccine hesitancy etc. Were their survey findings that could corroborate these findings. It was compared to previous research but this seemed out of place if it was comparing with survey findings from other quant research. This was not a major problem of the paper but maybe some attention or at least acknowledged as a limitation. | These data were only from a ‘content analysis’ of the themes. The survey data are being analysed and reported separately and do not cover all the themes considered; also just as the content analysis has proved to be, it would probably be too much for the paper. However I found the content analysis helpful when ensuring accurate coverage of what was a very large dataset for thematic analysis. Given that the tables and references to their data are confusing and perhaps also make for a less easy read, I have removed them (unless reference to the numbers was helpful). There were sufficient for some statistical analysis but given that sampling was purposive quota-based this would not be appropriate, and comparisons were made carefully so as not to suggest a greater confidence in them than was possible given the sampling approach. |
| Similarly, in places during the results there were some lengthy quotes and in some places 3 or 4 quotes where 2 would probably have been sufficient. All the material was fine and the quotes were easy to read but it seemed like the paper could have been shortened in length. | I have removed some repetitive quotes and followed a reviewer 2 comment that some could be shortened and added to the text. |
| Line 107, check grammar. | Word added for clarity |
| **Reviewer 2:** |  |
| The manuscript underscores the significance of incorporating viewpoints from society's most vulnerable demographics, including individuals with disabilities, racial and ethnic minorities, and undocumented individuals. I appreciated reading how the analysis and interpretation of findings integrated an intersectional approach. The methods section offered a clear and comprehensive overview of the qualitative study's methodology process. | Many thanks |
| However, due to the extensive number of interviews and collected data, the manuscript appears overly lengthy. Authors should contemplate integrating selected quotes into relevant paragraphs where they bolster major points, instead of appending them at the paragraph's end. This can help condensed manuscript. Additionally, certain themes could be merged together. | I have taken up this suggestion to achieve some text reduction and improve some arguments, thank you.  I was also surprised to realise that some themes were mentioned at different points as if not mentioned before, so thank you for spotting this, which has also helped reduce the manuscript. I believe the arguments are now much tighter. |
| Prior to resubmission, it's advisable to seek input from seasoned qualitative researchers to review the results and discussion sections thoroughly. | I hope the manuscript is now satisfactory? We are all seasoned qualitative researchers, but this was a group effort leading to more repetition and poorly integrated theme work than I had realised on submission. Shortening the paper has made this easier for me to spot (!) so I hope that these errors have all been dealt with. |
